# Forest Gaps Alter the Total Phenol Dynamics in Decomposing Litter in an Alpine Fir Forest

**DOI:** 10.1371/journal.pone.0148426

**Published:** 2016-02-05

**Authors:** Han Li, Liya Xu, Fuzhong Wu, Wanqin Yang, Xiangyin Ni, Jie He, Bo Tan, Yi Hu

**Affiliations:** 1 Long-term Research Station of Alpine Forest Ecosystems, Institute of Ecology & Forestry, Sichuan Agricultural University, Chengdu, 611130, China; 2 Collaborative Innovation Center of Ecological Security in the Upper Reaches of Yangtze River, Chengdu, 611130, China; 3 Sichuan Technology and Business University. International Business School, No. 9 Min River Eastern Section, Meishan City, China; Chinese Academy of Sciences, CHINA

## Abstract

The total phenol content in decomposing litter not only acts as a crucial litter quality indicator, but is also closely related to litter humification due to its tight absorption to clay particles. However, limited attention has been focused on the total phenol dynamics in foliar litter in relation to forest gaps. Here, the foliar litter of six representative tree species was incubated on the forest floor from the gap center to the closed canopy of an alpine Minjiang fir (*Abies faxoniana*) forest in the upper reaches of the Yangtze River and eastern Tibetan Plateau. The dynamics of total phenol concentration in the incubated litter was measured from November 2012 to October 2014. Over two-year incubation, 78.22% to 94.06% of total phenols were lost from the foliar litter, but 52.08% to 86.41% of this occurred in the first year. Forest gaps accelerated the loss of total phenols in the foliar litter in the winter, although they inhibited the loss of total phenols during the growing season in the first year. In comparison with the effects of forest gaps, the variations of litter quality among different species were much stronger on the dynamics of total phenols in the second year. Overall, the loss of total phenols in the foliar litter was slightly higher in both the canopy gap and the expanded gap than in the gap center and under the closed canopy. The results suggest that the predicted decline in snow cover resulting from winter warming or vanishing gaps caused by forest regeneration will retard the loss of total phenol content in the foliar litter of alpine forest ecosystems, especially in the first decomposition year.

## Introduction

Foliar litter is an important source of nutrients and energy inputs in forest ecosystems, and litter decomposition enriches the soil nutrient pool and supports ecosystem productivity [1,2]. Total phenols, mainly occurring as secondary metabolites, accounts for approximately 25% of the leaf dry mass [3] and includes monopolymer flavonoids, phenolic acids and polymer tannins [4]. These phenols in foliar litter can enter and tightly absorb to clay particles, which results in the formation of humus and an accumulation of organic matter and nutrients in the soil [3,5,6]. Total phenols in litter also act as a crucial indicator of changes in litter quality because they can inhibit microbial activity and precipitate protein in litter [7,8]. Therefore, understanding the dynamics of total phenol during litter decomposition can help identify litter decomposition mechanisms in forest ecosystems. However, the dynamics of the total phenols in foliar litter remains poorly understood.

In previous studies, rapid losses of the total phenols in foliar litter have been demonstrated in the early decomposition stages after leaf fall, which tends to be closely related to eluviation because of precipitation or snowmelt [9,10]. The changes following leaf fall in the total phenols of litterlargely occur via two main routes: 1) complexation with leaf protein [11] and plant cell-wall polysaccharides [12], and 2) biodegradation and oxidation [8,13,14]. Therefore, the total phenol dynamics in foliar litter are susceptible to environmental factors, litter quality and microbial decomposers that vary with the season. With the cold climate of the winter, the seasonal snowpack which is formed throughout the late autumn, winter and early spring, maintains microbial community abundance and activity, which are beneficial for accelerating biodegradation [15,16]. In addition, dramatic leaching caused by the melting of deep snow patches is expected to significantly alter the loss of litter total phenols. However, information on the effects of seasonal snowpack on the loss of total phenol content in decomposing litter is unavailable.

As a basic mechanism of forest regeneration and succession, gaps resulting from various forest disturbances are widely distributed in primary forests worldwide [17,18]. Gap formation not only changes the plant diversity of the forest ecosystem inside and outside of the gap [17] but also alters the decomposition environment by redistributing precipitation and light and altering the decomposer community via changes to the substrate quality and hydrothermal dynamics in the soil [19,20]. Based on surface atmospheric conditions, radiation, snowpack, soil thermal state and soil unfrozen water content [21], four critical periods (snow formation period, snow cover period, snow melt period and growing season) can be identified during one year of litter decomposition in regions with seasonal snow cover. With the cold climate of the winter, increasing snow cover depths have been observed from the closed canopy to the gap center because of canopy interception [22,23], which leads to significant differences in hydrothermal and microbial conditions among gap positions. The deep snow patches and long-term coverage at the center of the gap maintain the abundance and activity of the microbial community and accelerate biodegradation during the snow formation and coverage period, as well as leaching during the snow melt period [15,16]. However, because of the effects of canopy shading, a suitable decomposition environment with mild light is maintained, and sufficient summer rainfall in the closed canopy promotes the loss of total phenol contents in the growing season [19,22]. Previous studies have primarily focused on the mass loss, nutrient release and microbial biomass in decomposing litter in alpine forests [23–25]; however, information on the progress of total phenol decomposition in the litter in different forest gap positions is rare. We hypothesized that the total phenol decomposition in litter would decrease from the center of the forest gap to the closed canopy in the winter and that this trend would be reversed in the growing season.

To test this hypothesis, a field experiment was performed to determine the litter decomposition of four tree species (Minjiang fir (*Abies faxoniana*), cypress (*Sabina saltuaria*), Masters larch (*Larix mastersiana*), and red birch (*Betula albosinensis*)) and two shrub species (Kangding willow (*Salix paraplesia*), and Lapland azalea (*Rhododendron lapponicum*)) in a Minjiang fir alpine forest located along the upper reaches of the Yangtze River and the eastern Tibetan Plateau. As a vital role in stocking and preserving the soil and headwater quality, this type of forest exhibits long-term snow coverage with pronounced snowpack gradients from the gap center to the closed canopy as well as frequent soil freeze-thaw cycles in the winter. Furthermore, the forest of the present study redistributes precipitation and solar radiation in the growing season [26–28]. Previous studies have indicated that forest gaps markedly accelerate litter decomposition during the winter [23,29]. In this study, we measured the total phenol concentration and loss from six decomposing litters at critical periods during the winter and growing seasons over two years using the Folin Ciocalteau method. Our experimental results provide a more comprehensive understanding of the mechanisms underlying carbon and nutrient cycling in alpine forest ecosystems.

## Materials and Methods

### Site description

A litterbag study was conducted in the Miyaluo Nature Reserve, Li County, Sichuan Province, Southwestern China (latitude 31°14′–31°19′ N, longitude 102°53′–102°57′ E, altitude 2458 ~ 4619 m above MSL). The reserve is a transitional area between the Tibetan Plateau and the Sichuan Basin and is located along the upper reaches of the Yangtze River and the eastern Tibetan Plateau [25]. The mean annual air temperature and the mean annual rainfall at the site are 2.7°C and 850 mm, respectively, and the mean temperature in January and July is -8.7°C and 9.5°C, respectively. The winter typically begins in late October and lasts until April, presenting marked soil freeze-thaw cycles [30]. The tree canopy is dominated by *Abies faxoniana*, *Betula albosinensis*, *Larix mastersiana* and *Sabina saltuaria*, and the understory shrub layer is dominated by species such as *Salix paraplesia*, *Rhododendron lapponicum*, *Berberis sargentiana*, *Sorbus rufopilosa* and *Hippophae rhamnoides*. The herb layer is dominated by *Parasenecio forrestii*, *Cystopteris fragilis* and varieties of mosses [29].

### Field experiment

**Fig 1:**
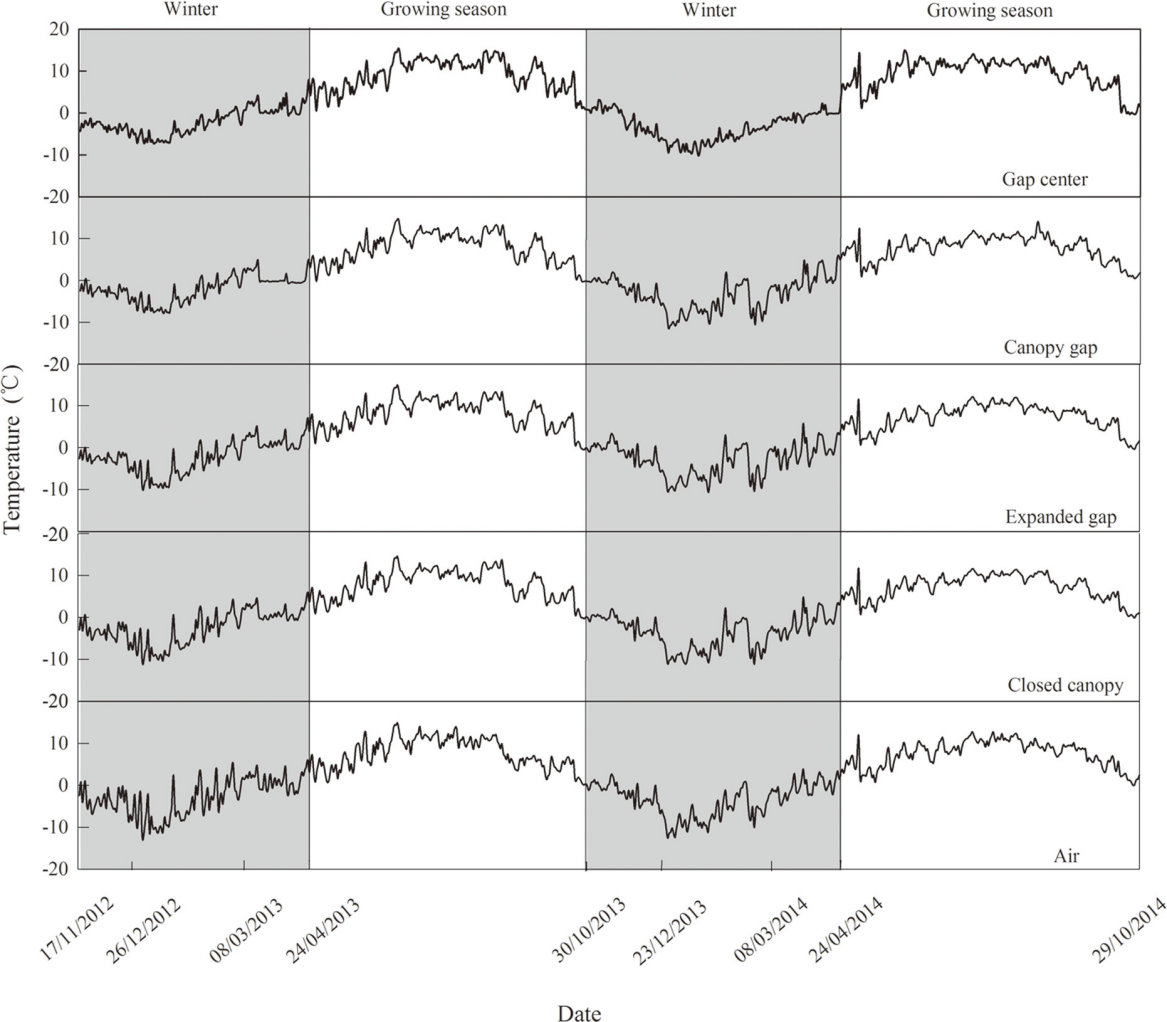
Daily mean air and soil surface temperatures of the four gap positions (gap center, canopy gap, expanded gap and closed gap) during two years of litter decomposition in an alpine Minjiang fir forest (November 17, 2012 to October 29, 2014) (n = 6).

The decomposition of six types of foliar litter (Minjiang fir, cypress, Masters larch, red birch, Kangding willow, Lapland azalea) was studied using the standard mesh bag technique. Freshly fallen litter of each species was collected from the forest floor of the study site in early October 2012 and air dried for two weeks in shade to prepare the litter material for decomposition. Approximately 10.00 g of air-dried material was placed within 20 × 25 cm nylon litterbags (mesh sizes 1.0 mm above and 0.5 mm below), and 288 bags were prepared for each species. Based on field investigation and previously collected local data, three forest gaps larger than 25 × 25 m and of similar canopy densities were randomly selected within a representative Minjiang fir forest (latitude 31°14′N, longitude 102°53′E; altitude 3579 ~ 3582 m above MSL) in the reserve. Four positions within each gap were established from the center of the gap to an area of closed canopy (gap center, canopy gap, expanded gap, and closed canopy). The bags were randomly staked onto the surface of the soil in blocks marked for each species (2 × 2 m) on 17 November, 2012. To avoid overvaluing the initial litter mass, which could occur due to litter falling out of litter bags in the course of transportation, we also sampled the initial litter bags from the study site on 17 November, 2012 and treated this litter remaining mass as the initial litter mass. According to Olsson’s period division of the cold season [21]and field investigations in our previous studies [22,23], samples were collected at the end of each critical period in two consecutive years: 26 December, 2012, the first snow formation period (SF1); 8 March, 2013, the first snow cover period (SC1); 24 April, 2013, the first snow melt period (SM1); 30 October, 2013, the first growing season (GS1); 23 December, 2013, the second snow formation period (SF2); 10 March, 2014, the second snow cover period (SC2); 24 April, 2014, the second snow melt period (SM2); and 29 October, 2014, the second growing season (GS2) (Fig 1). Three bags were retrieved from each species in each gap position at each sampling event and immediately transported to the laboratory.

**Fig 2:**
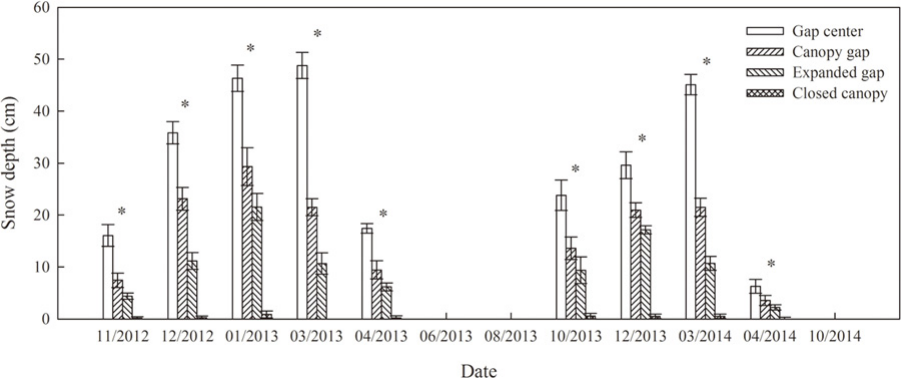
Snow depth (cm) of the four gap positions (gap center, canopy gap, expanded gap and closed canopy) during two years of litter decomposition in an alpine Minjiang fir forest (n = 3).

The temperature of the soil surface and air were automatically measured every two hours using iButton recorders (iButton DS1923-F5, Maxim/Dallas Semiconductor, Sunnyvale, CA, USA). The recorders were placed in two litterbags at each gap position and hung approximately 2 m from the forest floor in each plot. Because of the harsh environmental conditions and a lack of electrical power at the study site, the depth of the snow cover could not be monitored in real time; therefore, it was measured at multiple points with a ruler during each sampling event. Figs 1 and 2 show the temperature and snow cover depth data, respectively, over the two years of litter decomposition.

### Laboratory analyses of initial quality and total phenol content of the litter

**Fig 3:**
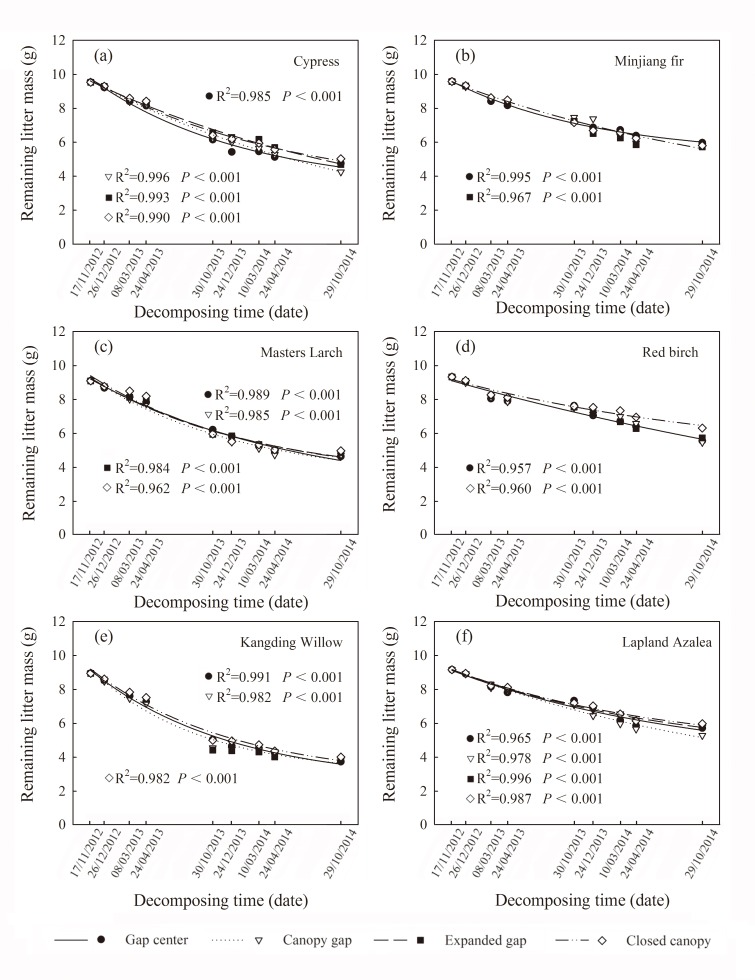
Exponential regression of the litter remaining mass (g) from the six types of foliar litter (cypress, Minjiang fir, Masters larch, red birch, Kangding willow and Lapland azalea) in the four gap positions (gap center, canopy gap, expanded gap and closed canopy) at each sampling event over two years of decomposition in an alpine Minjiang fir forest (mean, n = 3).

The initial samples of litter material of the six species from 17 November, 2012 and litter samples retrieved at the end of each critical period were oven dried at 65°C for 48 h, weighed to obtain the remaining mass (Fig 3), and then finely ground to pass through a 0.25 mm mesh. The initial concentrations of organic carbon (C), total nitrogen (N), and total phosphorus (P) were determined by the dichromate oxidation method, the Kjeldahl method (KDN, Top Ltd., Zhejiang, China), phosphomolybdenum yellow spectrophotometry (TU-1901, Puxi Ltd., Beijing, China), respectively[31,32]. Cellulose and lignin in the foliar litter were measured using the Van Soest method [33]. This method yields cellulose and acid-detergent lignin (AD lignin), which has recently been referred to as AUR (Acid Unhydrolyzable Residue) [34,35] (Table 1). The total phenol concentration was measured using the Folin Ciocalteau method [8,36]. Total phenol was extracted from the samples of oven-dried litter. In brief, the 50 mg samples were extracted in 15 ml of 70% acetone [acetone-water, 70:30, v/v] using ultrasonic wave extraction, and then centrifuged at 4°C and 5000 r/min for 10 min. Next, the supernatant liquid was mixed well with Folin-Ciocalteau reagent and sodium carbonate solution and allowed to react in darkness for 40 min, and the absorbance was measured at 725 nm. All of the analyses were carried out in triplicate.

**Table 1:** Initial quality of the six types of foliar litter (cypress, Minjiang fir, Masters larch, red birch, Kangding willow and Lapland azalea) in an alpine Minjiang fir forest (mean ± SD, n = 3). Different lowercase letters indicate a significant difference among species within the same variable (one-way ANOVA, *P* < 0.05).

Species	Total phenol(mg/g)	C(%)	N(%)	P(%)	AD lignin(%)	Cellulose(%)	C/N	C/P	N/P	AD lignin/N
Cypress	24.98(1.33)^c^	51.64(1.77)^bc^	0.88(0.01)^b^	0.12(0.01)^cd^	14.07(0.74)^b^	12.22(0.38)^a^	58.86(2.21)^b^	416.02(14.04)^a^	7.08(0.41)^ab^	16.04(1.01)^a^
Minjiang fir	29.34(2.68)^d^	50.56(2.96)^b^	0.88(0.01)^b^	0.11(0.01)^bc^	15.85(0.36)^b^	12.19(0.20)^b^	57.77(3.53)^b^	443.51(36.69)^ab^	7.68(0.72)^b^	18.11(0.42)^b^
Masters larch	29.57(2.27)^d^	54.35 (0.63)^c^	0.86(0.04)^b^	0.13(0.01)^d^	27.21(2.21)^d^	16.45(0.44)^d^	63.32(3.49)^b^	407.08(2.42)^a^	6.44(0.38)^a^	25.95(1.08)^c^
Red birch	11.28(1.46)^a^	49.69(1.45)^b^	1.33(0.02)^d^	0.09(0.01)^a^	36.68(0.62)^b^	12.47(0.38)^e^	37.24(1.35)^a^	544.94(31.72)^c^	14.63(0.36)^d^	25.99(0.37)^c^
Kangding willow	19.60(0.70)^b^	45.23(1.65)^a^	1.15(0.03)^c^	0.11(0.00)^b^	20.22(0.22)^a^	10.45(0.59)^a^	39.49(2018)^a^	408.12(16.89)^a^	10.34(0.18)^c^	17.66(0.63)^b^
Lapland azalea	31.60(0.62)^d^	50.29(1.60)^b^	0.67(0.02)^a^	0.11(0.01)^b^	20.81(0.18)^c^	14.07(0.41)^c^	75.54(4047)^c^	471.14(42.04)^b^	6.25(0.25)^a^	31.24(0.69)^d^

### Statistical analysis and calculations

For the environmental factors, we calculated the mean daily temperature (MDT), positive accumulated temperature (PAT), negative accumulated temperature (NAT), positive degree days (PDD) (number of days that the mean daily temperature was above 0°C), negative degree days (NDD) (number of days that the mean daily temperature was below 0°C) and number of freeze-thaw cycles per day (FTC) (one freeze-thaw cycle was completed when the threshold of 0°C was crossed twice over a period of at least 3 [37]) of each critical period (Table 2). The total phenol loss was calculated as follows:

$$Loss\;(\%)=[(C_{t-1}\;\times \;M_{t-1})－(C_{t}\;\times \;M_{t})]\;/\;(C_{0}\;\times \;M_{0})\;\times \;100$$

where *M*_*t*_ and *M*_*t-1*_ represent the litter’s remaining dry mass (g) between the current and previous sampling dates, respectively; *C*_*t*_ and *C*_*t-1*_ represent the concentrations (%) of total phenol between the current and previous sampling dates, respectively; *M*_*0*_ represents the initial litter dry mass; and *C*_*0*_ represents the initial concentration (%) of total phenols.

**Table 2:** Characteristics of the mean daily temperature (MAT, °C), positive accumulated temperature (PAT, °C), negative accumulated temperature (NAT, °C), positive degree days (PDD, days), negative degree days (NDD, days) and freeze-thaw cycles per day (FTC, times) across each period in the four gap positions (gap center, canopy gap, expanded gap and closed gap) over two years of litter decomposition in an alpine Minjiang fir forest. The first snow formation period (SF1), the first snow cover period (SC1), the first snow melt period (SM1), the first growing season (GS1), the second snow formation period (SF2), the second snow cover period (SC2), the second snow melt period (SM2), and the second growing season (GS2).

Gap		1st year				2nd year			
position		SF1	SC1	SM1	GS1	SF2	SC2	SM2	GS2
	MDT	-3.55	-3.44	1.88	8.90	-3.07	-5.22	2.04	9.49
	PAT	0	14	91	1683	25	0	111	1775
Gap	NAT	-142	-262	-3	0	-191	-392	-15	-1
center	PDD	0	8	35	187	14	4	21	181
	NDD	39	63	10	2	40	71	18	4
	FTC	0.45	0.41	0.45	0.08	0.72	0.06	0.24	0.11
	MDT	-2.88	-3.14	0.93	7.66	-3.66	-4.89	2.91	7.75
	PAT	1	24	53	1451	7	4	153	1452
Canopy	NAT	-116	-250	-9	-2	-205	-371	-16	-2
gap	PDD	1	15	17	176	7	4	32	178
	NDD	39	57	25	7	47	70	15	6
	FTC	0.59	0.61	0.26	0.10	0.67	0.62	0.56	0.08
	MDT	-3.87	-4.20	1.94	7.87	-2.91	-4.52	1.71	7.38
	PAT	0	17	93	1493	22	12	113	1383
Expanded	NAT	-117	-320	-2	-5	-179	-433	-32	-2
gap	PDD	1	11	33	183	12	9	28	184
	NDD	39	61	10	6	42	65	18	3
	FTC	0.52	0.53	0.55	0.09	0.69	0.60	0.53	0.08
	MDT	-4.11	-4.05	1.51	7.70	-3.30	-5.75	1.54	7.36
	PAT	0	24	79	1458	8	2	108	1379
Closed	NAT	-155	-316	-8	-3	-186	-418	-35	-2
canopy	PDD	1	15	30	181	8	2	27	181
	NDD	39	57	16	6	46	73	16	4
	FTC	0.63	0.59	0.48	0.04	0.60	0.39	0.46	0.07

The differences in snow cover depth, the initial quality of the six types of litter, the total phenol concentration in each sampling date and the total phenol loss among the four gap positions of the six litters at each period over the two years were evaluated using one-way ANOVA (analysis of variance) and LSD (least significant difference) test at the 0.05 level. After pairwise comparison tests using MANOVA (multivariate analysis of variance), the responses of the variables (remaining mass and total phenol and loss) to the decomposition period across the gap positions were evaluated by single exponential regression or non-parametric LOESS regression with a 95% confidence interval (CI). The homogeneity of variance was tested using Levene’s test, and any data sets failing this test were log-transformed before further analysis to help satisfy the requirement of variance homogeneity. The effects of period, gap position and species on the total phenol loss in the six types of decomposing litter over the two years were evaluated using a repeated measures ANOVA (Table 3). The relationships between the total phenol loss and each of the environmental factors and litter quality factors were determined using Pearson’s correlation coefficient (Table 4). Additionally, the relationship between the total phenol concentration and remaining litter mass was calculated using Pearson’s correlation coefficients. The ANOVA, Person’s correlation and non-parametric LOESS regression analyses were implemented in the Statistical Product and Service Solutions program (SPSS version 21.0, IBM, USA), whereas the exponential and linear regressions were fitted using SigmaPlot 12.0 (Systat Software Inc., USA).

**Table 3:** Repeated measures ANOVA of the effects of period, gap position and species on litter total phenol loss of the litter over two years of litter decomposition in an alpine Minjiang fir forest. (n = 48).

Source variance	*df*	*F*-value	*P*-value
Period (P)	12	623.989	<0.001**
Gap position (G)	3	6.202	0.001**
Species (S)	5	40.878	<0.001**
P×G	36	2.862	<0.001**
P×S	60	8.920	<0.001**
G×S	15	1.881	0.050*
P×G×S	180	1.592	<0.001**

* Significant at the 0.05 probability level.

** Significant at the 0.01 probability level.

**Table 4:** Pearson’s correlations (r) between litter total phenol loss of the litter and each of the environmental factors and litter quality factors at critical periods of six decomposing litters during two years in an alpine Minjiang fir forest. The first/second winter (W1/W2), the first/second growing season (G(S)1/G(S)2), mean daily temperature (MDT), positive accumulated temperature (PAT), negative accumulated temperature (NAT), positive degree days (PDD), negative degree days (NDD), freeze-thaw cycles per day (FTC).

Source variance		Correlation coefficient						
		W1	G1	W2	G2	1st year	2nd year	Two years
	Initial total phenol	0.016	0.113	0.329**	-0.358**	0.142	-0.040	0.269**
	Initial C	-0.032	-0.093	0.061	0.046	-0.141	0.097	-0.183
	Initial N	0.079	-0.100	-0.424**	0.316**	-0.004	-0.082	-0.133
	Initial P	0.377**	0.072	-0.017	-0.537**	0.569**	-0.511**	0.555**
Litter quality	Initial AD lignin	0.123	0.002	0.150	-0.312**	0.163	-0.156	0.144
factors	Initial cellulose	-0.135	-0.272**	-0.245*	0.580**	-0.468**	0.319**	-0.607**
	Initial C/N	-0.145	0.022	0.424**	-0.177	-0.166	0.211	-0.069
	Initial C/P	-0.457**	-0.189	0.079	0.680**	-0.799**	0.698**	-0.810**
	Initial N/P	-0.096	-0.143	-0.299**	0.482**	-0.278*	0.181	-0.374**
	Initial AD lignin/N	-0.228	-0.243*	0.186	0.310**	-0.557**	0.452**	-0.617**
	MDT	0.113	-0.214	0.061	0.229	-0.048	-0.007	-0.081
	PAT	-0.162	-0.215	0.086	0.229	-0.045	-0.039	-0.117
Environmental	NAT	0.223	-0.097	0.065	0.059	-0.020	0.084	0.048
factors	PDD	-0.237*	0.021	0.139	0.014	-0.064	0.089	-0.074
	NDD	0.195	0.019	-0.009	-0.008	0.065	-0.061	0.064
	FTC	-0.092	-0.175	0.326**	0.108	-0.018	0.117	0.127

* Significant at the 0.05 probability level.

** Significant at the 0.01 probability level.

## Results

### Litter initial quality and remaining mass

The concentrations of total phenols, C, N, P, AD lignin and cellulose as well as the ratios of C/N, C/P, N/P and AD lignin/N in the initial litter varied markedly among the six species (Table 1). The initial total phenol concentrations of the six types of litter ranged from 11.28% to 31.60%. The Lapland azalea leaf litter had the largest total phenol concentration and AD lignin/N ratio and the smallest N concentration, whereas the red birch foliar litter had the smallest total phenol, P, N and AD lignin concentrations as well as the largest ratios for C/P and N/P. According to the results of the single exponential regression, the litter remaining mass of all the six types of litter showed a decline over the two years of decomposition from 8.94 ~ 9.58 g to 3.74 ~ 6.32 g. Kangding willow and Lapland azalea litter exhibited the maximum and minimum mass losses, respectively. Compared with the other gap positions, the closed canopy exhibited a significantly larger remaining mass of foliar litter (Fig 3). Moreover, the total phenol concentration was significantly and positively related to the remaining litter mass (Pearson’s r = 0.767, *P* < 0.001).

### Effects of forest gaps on the total phenol concentration of litter

**Fig 4:**
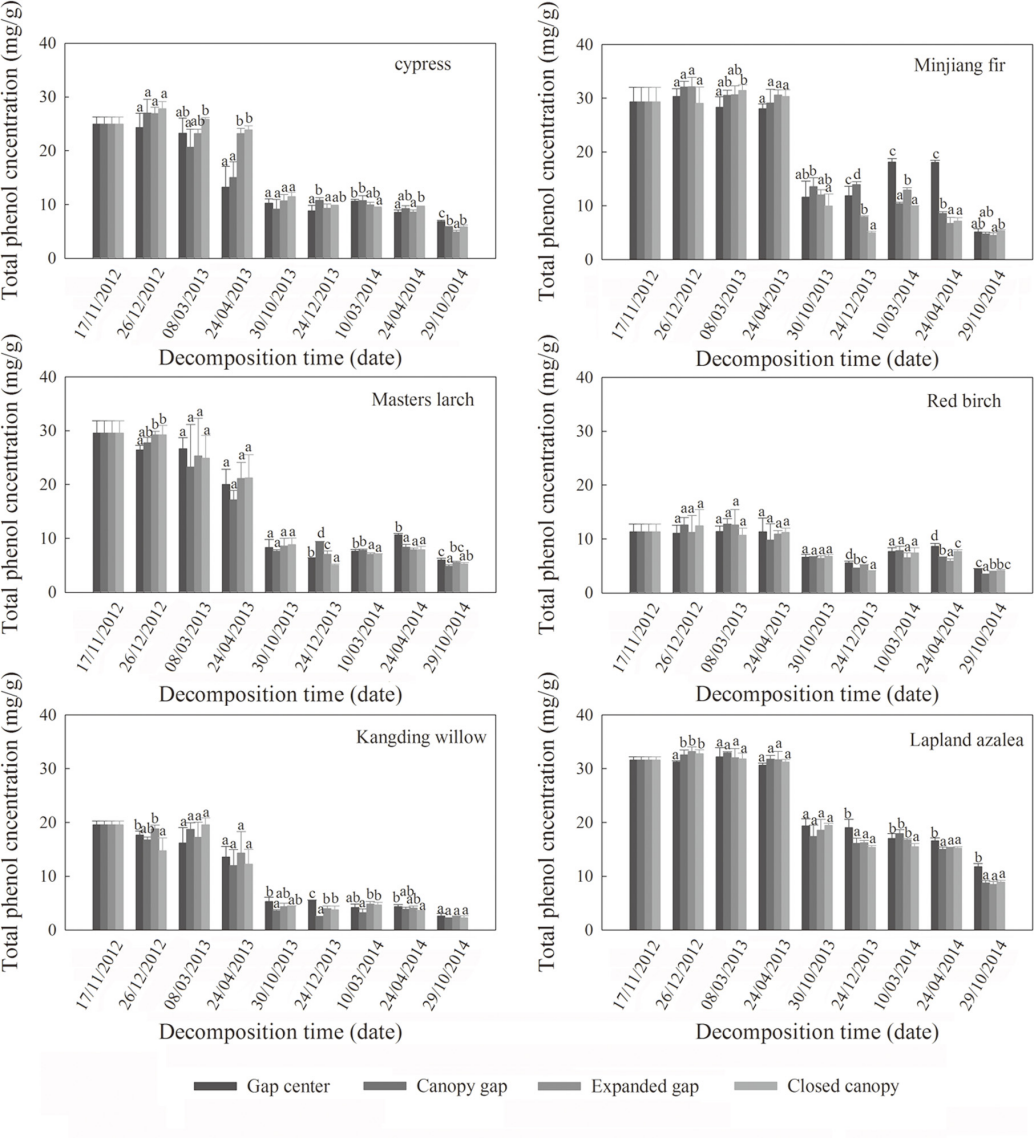
Total phenol concentration (mg/g) of the six types of foliar litter (cypress, Minjiang fir, Masters larch, red birch, Kangding willow and Lapland azalea) in the four gap positions (gap center, canopy gap, expanded gap and closed canopy) at each sampling date over two years of decomposition in an alpine Minjiang fir forest. Different lowercase letters indicate significant differences among gap positions at the same sampling time, *P* < 0.05 (mean ± SD, n = 3).

As shown in Fig 4, total phenol concentration declined consistently in all six types of litter over the two years of litter decomposition. There was a rapid decrease in total phenol concentration during the first year of decomposition regardless of species, whereas a gradual decrease occurred in the second year. The changes in total phenol concentration at each critical period were more pronounced in the growing seasons than in the other decomposition periods of the winter, especially in the first growing season. During the first year, litter total phenol exhibited lower concentrations in the gap center and canopy gap compared with the expanded gap and closed canopy with the exception of the total phenol in red bitch litter, in which no significant differences among gap positions were observed. In the second year, the trend was reversed such that the total phenol concentration in litter was higher in the gap center and the canopy gap than in the expanded gap and the closed canopy, especially at the end of the second snow cover and melt period.

### Effects of forest gaps on the total phenol loss of litter

**Fig 5:**
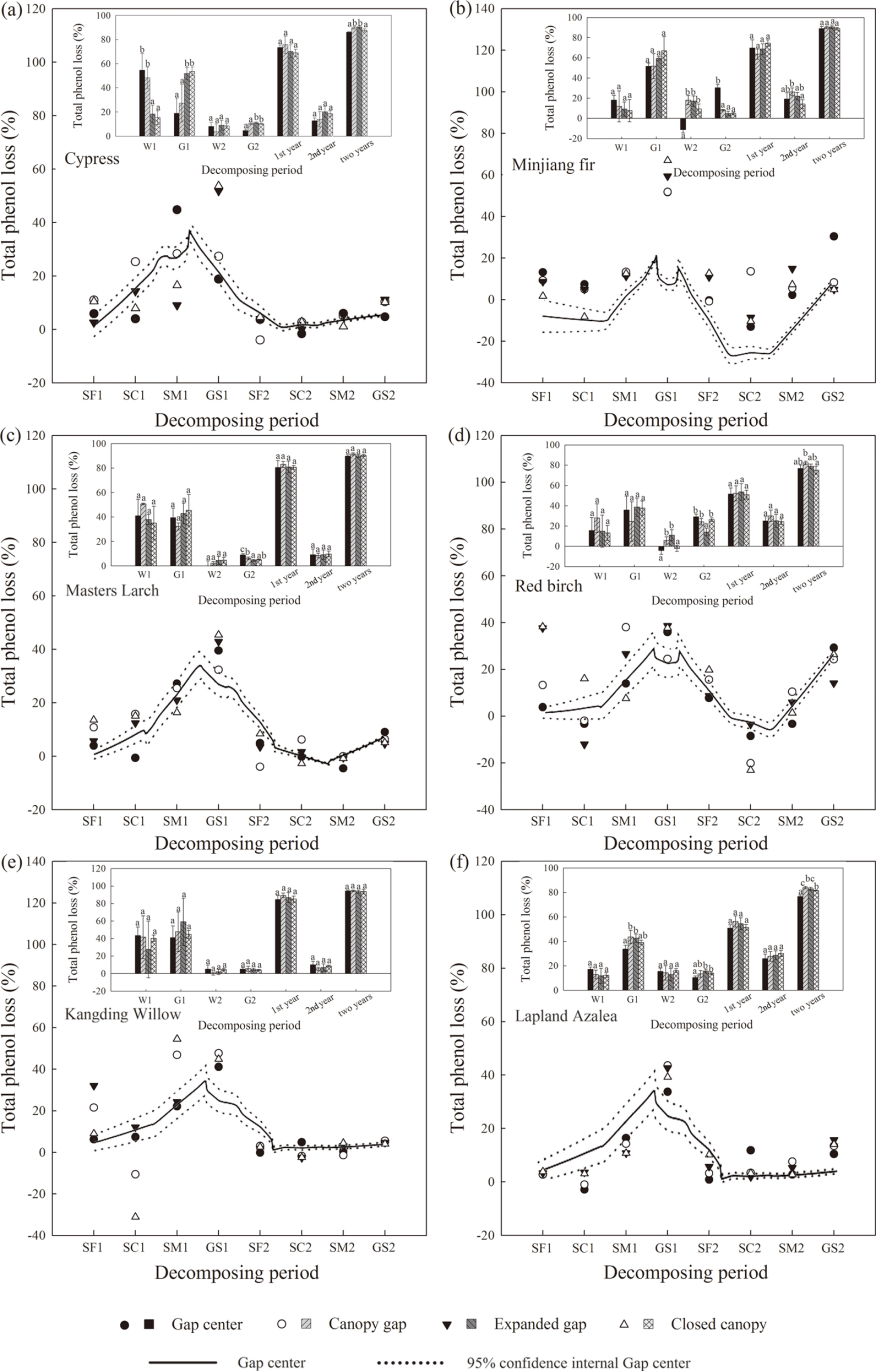
Total phenol loss (%) of the six types of foliar litter (cypress, Minjiang fir, Masters larch, red birch, Kangding willow and Lapland azalea) in the four gap positions (gap center, canopy gap, expanded gap and closed canopy) at the end of each period over two years of decomposition in an alpine Minjiang fir forest. The first/second snow formation period (SF1/SF2), the first/second snow cover period (SC1/SC2), the first/second snow melt period (SM1/SM2), the first/second winter (W1/W2), the first/second growing season (G(S)1/G(S)2), the first/second year of decomposition (1st/2nd year), two years of litter decomposition (two years) Columns marked with different lowercase letters indicate significant differences among gap positions within the same period, *P* < 0.05 (mean ± 95% confidence intervals or mean ± SD, n = 3).

Total phenol losses in litter across the two years of decomposition were high in all of the six types of litter and ranged from 78.22% to 94.06% (Fig 5) depending on species, gap position and period (Table 3). Cypress, Masters larch and Kangding willow litter had high total phenol losses of approximately 90% after two years, and more rapid total phenol loss was observed during the first year than in the second, with similarly high total phenol losses in the first winter and growing season. However, the total phenol losses in the first growing season were higher compared with those in the first winter in Minjiang fir, red birch and Lapland azalea litter, which showed marked total phenol losses (20.43 ~ 28.06%) in the second growing season. Considerable increases in total phenol loss occurred in all six types of litter across each critical period of the first year, with a peak occurring in the first growing season and a pronounced decrease in the second snow formation period. Subsequently, the total phenol content increased slightly in the cypress, Masters larch, Kangding willow and Lapland azalea litter and sharply increased in the Minjiang fir and red birch litter from the second snow cover period to the second growing season. Higher total phenol losses in all species tended to occur at the gap center in the winter and at the closed canopy in the growing season during the first year of litter decomposition. The Minjiang fir and red birch litter exhibited markedly higher total phenol losses at the canopy gap and expanded gap during the second winter, whereas the effects of forest gaps on total phenol loss in the other species were not significant. Over the second growing season, the highest total phenol losses were obtained at the gap center for the Minjiang fir, Masters larch and red birch litter and at the canopy gap and closed canopy for the cypress and Lapland azalea litter. Furthermore, stronger significant correlations were observed between the quality factors and total phenol loss, especially in the second year, although the PDD and FTC tended to show a significant negative and positive relationship with total phenol loss, respectively (Table 4).

## Discussion

During the first year of litter decomposition, the hypothesis that the decomposition of total phenol of the litter would decrease from the forest gap center to the closed canopy in the winter and increase in the growing season was partially supported. However, in the second year, inconsistencies with this pattern emerged in the winter and the growing season along with observed interspecific differences. In the present study, the ambient environmental conditions in each gap position remained the same for the six species; therefore, variation in the total phenol decomposition of the litter may have been caused by the litter material. Moreover, the incremental correlations between the quality factors and the total phenol losses of the litter in the second year of decomposition indicate that litter quality might be a major factor governing the decomposition of total phenol content in the late period of litter decomposition. The results revealed that forest gaps promoted and inhibited total phenol decomposition during the first winter and growing season, respectively, whereas different influences were observed in the second year due to interspecific differences.

The changes that occurred in total phenol in the six litter species during the two winters varied with gap position and species. The absence of detected phenolic compounds in the soil leachate and the rapid loss from fallen leaf litter reported in previous studies [8,38] suggest that total phenol in the present study was primarily lost through leaching and then tightly absorbed by the soil during the first winter. As the depth of snow coverage decreased, the increased leaching at the gap center resulted in the highest loss of total phenol content regardless of species, especially in the snow melt period [39]. Although there were similar gradient of the physical environmental conditions during the second winter as in the first, significant effects of these conditions on total phenol loss were not observed for the cypress, Masters larch, Kangding willow and Lapland azalea litter in the forest gaps. After an initial rapid loss of components with lower molecular weights, such as catechins, phenolic compounds may condense into less soluble forms or react with larger molecules, with the extent of increase dependent on microbial decomposer activity and physical breakdown during freeze-thaw cycles [2]. Therefore, generally low levels of microbial abundance and activity and the daily frequency of freeze-thaw cycles during the winter might account for the observed lower and undifferentiated total phenol losses in the litter in the four gap positions over the second winter [40]. Compared with the other periods, the snow cover period in each winter exhibited slightly lower total phenol losses in the six types of litter and even showed an accumulation of total phenol content at the gap center. These results, may be explained by the increased biodegradation that occurs in the largely stable ambient environment that results from the deep snow isolation and the subsequent increased amount of degradation intermediates of the phenolics (e.g., protocatechuic, vanillic acids) in the litter [2,5].

Previous studies have reported that rapid losses of total phenol continued for approximately two months in the early stage after leaf fall [9,10]; therefore, the subsequent changes in the total phenol of the litter in the growing seasons were mainly attributed to complexation with leaf proteins [11] or plant cell-wall polysaccharides [12] and biodegradation or oxidation, especially in the second growing season [8,13,14]. During the growing season, intensive solar radiation and evaporation tend to restrict the effectiveness and efficiency of microorganisms at the gap center. In contrast, the closed canopy during the growing season is characterized by mild hydrothermal conditions [28]. These characteristics of the gap center and closed canopy are consistent with the results of this study as the total phenol losses of the litter were found to decline from the closed canopy to the gap center in the first growing season. The loss of tannins from the leaf litter and the subsequent increases of toxic soil-absorbed tannins may have an effect on iron and nitrogen availability and inhibit biodegradation [41,42]. Because of the complex interaction between microenvironmental regulation and litter material late in the second growing season, the six species did not exhibit a consistent trend of total phenol losses in the four gap positions, with higher losses observed in the gap center for Minjiang fir, Masters larch, red birch and Kangding willow litter and in the expanded gap and closed canopy for cypress and Lapland azalea litter. Unlike in the first growing season, in which no significant differences in total phenol loss among species were observed due to the much stronger influence of environmental factors than of litter quality and in which litter decomposition was still at an early stage, interspecific differences in the second growing season were evidenced by the incremental correlations between litter total phenol loss and the initial quality of the litter.

Both the repeated measure ANOVA and the Pearson’s correlation coefficients revealed a significant effect of species on total phenol loss over the two years of the experiment. Litter decomposition generally follows a sequential pattern, with different classes of organic compounds dominating the decay process as it proceeds. The leaching of soluble and low-molecular-weight compounds, such as phenolic compounds, dominates the first stage of litter decay, whereas hemicellulose, cellulose and AD lignin degradation becomes dominant later [2,43]. Thus, the total phenol losses of the litter were much higher in the first year than in the second in our research. However, interspecific differences caused greater variability in the total phenol losses between the two years in the cypress (55.72%), Masters larch (72.02%) and Kangding willow (78.76%) litter compared with the Minjiang fir (48.80%), red birch (25.86%) and Lapland azalea (25.22%) litter. Additionally, the former three litters exhibited similar total phenol losses between the winter and growing seasons of each year, whereas the latter three types of litter exhibited more rapid total phenol losses in the growing season. Inhibition caused by more resistant components in the latter three litters than in the former litters, such as wax and cutin, may explain the long latency with low total phenol loss during the first winter [44], and the accelerated losses in the following growing season may have been caused by an improvement in litter quality that resulted from physical decomposition [29]. Over the entire two years, slightly higher total phenol losses were observed in the litter in the canopy gap and expanded canopy compared with those under the closed canopy and in gap center, which indicates that the intermediate hydrothermal conditions were beneficial to the long-term decomposition of the total phenol.

## Conclusions

This study describes the characteristics of total phenol decomposition for six typical types of foliar litter in an alpine Minjiang fir forest in relation to season. Over the two years of litter decomposition, all of the six types of foliar litter lost large amounts of total phenols, with values ranging from 78.22% to 94.06%.A higher total phenol loss was observed in the first year than in the second, especially in the first growing season. Effects of forest gaps were also mainly detected in the first year, and they accelerated the total phenol losses of the litter in the winter and inhibited phenol losses in the growing season. Although significant differences in total phenol loss were not observed among the four gap positions in the second year, stronger effects of litter quality were observed in the second year. Our results indicate that the total phenol dynamics of litter vary between the winter and growing season, and they also suggest that the predicted reductions in snow depth resulting from winter warming or the loss of forest gaps because of forest regeneration will inhibit the total phenol decomposition of litter in alpine forest ecosystems.

## Supporting Information

S1 File Original data of the study. (Temperature, snow cover, litter remaing mass and litter total phenol concentration).
(XLS)
